# Identification and experimental validation of autophagy-related genes in abdominal aortic aneurysm

**DOI:** 10.1186/s40001-023-01354-6

**Published:** 2023-09-22

**Authors:** Xiaoli Yuan, Yancheng Song, Hai Xin, Lu Zhang, Bingyu Liu, Jianmin Ma, Ruicong Sun, Xiaomei Guan, Zhirong Jiang

**Affiliations:** 1 Department of Cardiac Ultrasound, The Affiliated Hospital of Qingdao University, Qingdao, China; 2 Department of Gastrointestinal Surgery, The Affiliated Hospital of Qingdao University, Qingdao, China; 3 Department of Vascular Surgery, The Affiliated Hospital of Qingdao University, Qingdao, China

**Keywords:** Autophagy, AAA, Bioinformatics analysis, Gene expression omnibus dataset

## Abstract

**Aim:**

Autophagy plays essential roles in abdominal aortic aneurysm (AAA) development and progression. The objective of this study was to verify the autophagy-related genes (ARGs) underlying AAA empirically and using bioinformatics analysis.

**Methods:**

Two gene expression profile datasets GSE98278 and GSE57691 were downloaded from the Gene Expression Omnibus (GEO) database, and principal component analysis was performed. Following, the R software (version 4.0.0) was employed to analyze potentially differentially expressed genes related with AAA and autophagy. Subsequently, the candidate genes were screened using protein–protein interaction (PPI), gene ontology (GO) enrichment analysis, and Kyoto Encyclopedia of Genes and Genomes (KEGG) enrichment analysis. Finally, quantitative real-time polymerase chain reaction (RT-qPCR) was performed to detect the RNA expression levels of the top five selected abnormal ARGs in clinical samples obtained from the normal and AAA patients.

**Results:**

According to the information contained (97 AAA patients and 10 healthy controls) in the two datasets, a total of 44 differentially expressed autophagy-related genes (6 up-regulated genes and 38 down-regulated genes) were screened. GO enrichment analysis of differentially expressed autophagy-related genes (DEARGs) demonstrated that some enrichment items were associated with inflammation, and PPI analysis indicated interaction between these genes. RT-qPCR results presented that the expression levels of IL6, PPARG, SOD1, and MAP1LC3B were in accordance with the bioinformatics prediction results acquired from the mRNA chip.

**Conclusion:**

Bioinformatics analysis identified 44 potential autophagy-related differentially expressed genes in AAA. Further verification by RT- qPCR presented that IL6, PPARG, SOD1, and MAP1LC3B may affect the development of AAA by regulating autophagy. These findings might help explain the pathogenesis of AAA and be helpful in its diagnosis and treatment.

## Introduction

Abdominal aortic aneurysm (AAA) is a fatal condition which threatens public health. It consists of local enlargement of the abdominal aorta with reduced vascular smooth muscle cells (VSMCs) in the vascular middle layer. AAA is a common form of aneurysm, but it is usually asymptomatic and rupture is often accidental and fatal, with a mortality rate of 80% or more [1]. Previous studies have shown that risk factors for AAA include genetic factors, advanced age, male sex, smoking, hypertension, hyperlipidemia, obesity, atherosclerosis and other vascular occlusives [2]. Unfortunately, there is no clinically established effective pharmacological approach to limit the progression of AAA or the risk of rupture in humans, surgical intervention being the only viable treatment [3]. Growing evidence indicates that the pathogenesis of AAA involves multiple biological functions, including chronic inflammation, cell proliferation, apoptosis, and autophagy [4–6], and that its regulatory mechanism is complex. Therefore, it is important to explore the pathogenesis of AAA and find key targets and markers for its diagnosis and treatment.

Autophagy is a conserved mechanism that transports damaged, denatured, or senescent proteins and organelles to lysosomes for digestion and degradation [7]. Meanwhile, autophagy is a factor in cardiovascular and a range of other diseases. For example, lncRNA CAIF alleviates myocardial infarction and protects cardiac tissue by regulating cardiac autophagy through the p53-cardiomyin axis [8]. In addition, Mir-214-3p directly targets vascular endothelial cell ATG5, reduces ox-LDL-induced autophagy, and regulates the progression of atherosclerosis [9]. Studies have shown that certain signaling pathways affect the biological functions of AAA through autophagy. Autophagy induced by AngII is modulated by JAK2/STAT3 and NF-κB signaling, which is inhibited by BP-1-102, thus affected the progression of AAA [6]. However, the autophagy-related genes involved in AAA remain largely unclear and require further study. The exploration of subclinical ARGs of AAA will offer new latent targets for clinical treatment of AAA.

With rapid development of next-generation sequencing technology, ARG-based signs have been used to evaluate and verify the differential expression of genes in different types of diseases [10, 11]. Recent research has identified the potential roles of characteristic ARGs in the diagnosis of systemic lupus erythematosus (SLE) and revealed the correlation between their expression and DNA methylation [11]. However, the role of ARGs in AAA has not been fully clarified.

In this study, we explored the differentially expressed genes related with autophagy in AAA by analyzing the GSE98278 and GSE57691 datasets in the GEO database. Firstly, we screened 44 candidate genes. Following, PPI analysis and GO enrichment analyses were employed on the candidate genes. Lastly, we further verified the key genes expression levels among the candidate genes in clinical samples with AAA using RT-qPCR. Our results found that IL6, PPARG, SOD1, and MAP1LC3B may influence the process of AAA by regulating autophagy. These will help deepen the understanding of AAA and provide an effective reference for clinical diagnosis and treatment.

## Materials and methods

### Autophagy-related genes datasets and microarray data

GSE98278 was obtained from the GPL10558 platform (Illumina HumanHT-12 V4.0 Expression beadchip), including 17 AAA patients with rupture, 15 moderate AAA patients (mean maximum active diameter: ≤ 55 mm), and 16 large AAA patients (mean maximum aortic diameter: > 70 mm). The GSE57691 dataset was derived from the GPL10558 platform (Illumina HumanHT-12 V4.0 Expression beadchip), including 20 patients with minor AAA (mean maximum aortic diameter: 54.3 ± 2.3 mm) and 29 patients with large AAA (mean maximum aortic diameter: 68.4 ± 14.3 mm). A total of 796 ARGs were obtained from the human autophagy modulators database (HAMdb: http://hamdb.scbdd.com/). Gene expression profiles GSE98278 and GSE57691 were selected from the GEO database (http://www.ncbi.nlm.nih.gov/) (Table 1). The attached research workflow is shown in the figure: bioinformatics analysis workflow.

**Table 1:** Characteristics of the two microarray datasets

GSE ID	Participants	Tissues	Year	Platform
GSE98278	48 AAA	Aortic tissue	2018	GPL10558 Illumina HumanHT-12 V4.0 expression beadchip
GSE57691	49 AAA and 10 control aortic	Aortic tissue	2015	GPL10558 Illumina HumanHT-12 V4.0 expression beadchip

### Differentially expressed autophagy-related genes (DEARGs)

To obtain a standardized representation matrix of microarray database, we download information from the dataset and annotate the probes according to the annotation file.

Principal component analysis (PCA) was used to check the repeatability of the data in the GSE, and SVA R was used to eliminate batch effects. The data were standardized using the “LIMMA” toolkit in the R software (version 4.0.0). The threshold for DEGs was adjusted as |log2(fold change)|≥ 1.0 and *P* < 0.05. The “heatmap” and “ggplot2” packages in R software (version 4.0.0) are used to draw the heatmap and volcano plot.

### PPI network analysis and identification of hub ARGs

Compared with other genes, hub genes are expected to be key functional genes. DEARGs were evaluated using the STRING database (http://www.string-db.org/), with confidence level > 0.4 as the norm for PPI analysis. The Cytoscape software (version 3.8.1) was used to strategized the PPI network and Cytohubba plug-ins to research pivotal nodes in the network [12]. The degree method was made to recognize the core genes in PPI. The top seven genes were classified as hub ARGs.

### Functional enrichment analysis

GO and KEGG pathway enrichment analyses were employed using the clusterProfiler toolkit in R software [13]. The GO database explains gene products in terms of molecular functions, biological processes, and cellular components of biology.

### Patients with AAA and healthy individuals

Five patients with AAA (case group) and five healthy persons (control group) admitted to The Affiliated Hospital of Qingdao University between October 2021 and May 2022 were selected. Diagnosis of AAA was made using the following criteria: permanent localized dilation of the arterial wall greater than 50% of the normal vessel diameter, definitive diagnosis by CT angiography (CTA), and AAA is usually diagnosed on the basis of an abdominal aortic diameter > 3 cm. This study was approved by the hospital's medical ethics committee. All participants provided written informed consent. Basic characteristics of the subjects included in this study are provided in Table 2.

**Table 2:** Basic characteristics of the subjects included in this study

Characteristics	Control (*n* = 5)	Patients (*n* = 5)
Age, years	60 ± 4.6	60 ± 5.2
Sex, male: female	2:3	4:1
Heart rate	64.25 ± 10.75	71.11 ± 11.13
Hypertension, *n* (%)	2 (40%)	4 (80%)
Hyperlipidemia, *n* (%)	1 (20%)	3 (60%)
Smoking, *n* (%)	2 (40%)	4 (80%)
Maximal AAA diameter, cm	–	3.5 ± 2

### Tissue total RNA isolation

1. The surgically removed diseased tissue (about 2.5 g) was washed three times with pre-cooled PBS, cut into pieces, and transferred into a 1.5 mL centrifuge tube. An appropriate amount of grinding steel balls and 1 mL Trizol lysate were added and ground in a tissue grinder for 5 min, and the liquid was transferred to a new centrifuge tube.
2. Add 200 μL of chloroform, shake for 15 s, and stand for 10 min.
3. Centrifugation at 4 ℃: 12000 RPM/15 min.
4. After centrifugation, take out the EP tube, which can be seen to be divided into three layers. Take about 400 mL of the upper liquid into the new EP tube, and add 400 mL of pre-cooled isopropanol, slowly invert and mix for several times, and then stand on ice for 10 min.
5. Centrifugation: 12,000 RPM/10min/4 ℃.
6. After centrifugation, take out the EP tube, observe whether there is precipitation, discard the supernatant, and be careful not to suck out the precipitation. Add 75% ethanol in 1 mL DEPC water configuration, fully clean the precipitate, and centrifuge at 4:12000 RPM/10min/4 ℃.
7. Repeat the previous step twice.
8. After centrifugation, take out the EP tube, absorb the residual ethanol as far as possible, and then put it on ice in the fume hood to dry.
9. Concentration measurement, when the ratio of A260/A280 is between 1.8 and 2.0, it indicates that the RNA purity is good without obvious pollution, and the experiment can be continued. The extracted RNA needs to be stored in the refrigerator at − 80 ℃.

### RNA extraction and quantitative real-time polymerase chain reaction (RT-qPCR)

Tissue from patients with AAA (*n* = 5) and healthy controls (*n* = 5) were extracted using TRIZOL reagent (Vazyme, Nanjing, China). Total RNA was reversely transcribed into cDNA according to the manufacturer's protocol (Vazyme, Nanjing, China). Quantitative RT-PCR was conducted using SYBR Green qPCR Mix (Yeasen, Shanghai, China) in an Agilent Technologies AriaMx Real-Time PCR(G8830A) with the following cycle conditions: 95 ℃ for 5 min, 95 ℃ for 10 s, and 60 ℃ for 30 s, over 40 cycles. GAPDH can be used as an internal reference for gene screening. Primer sequences for RT-qPCR (Table 3). The 2^−ΔΔCT^ method was used for statistical analysis [14, 15]. All data were averaged from three independent experiments.

**Table 3:** Primer sequences for RT-qPCR

Gene name	Sequences (5′ → 3′)
GAPDH forward	AAGAAGGTGGTGAAGCAGGC
GAPDH reverse	TCCACCACCCAGTTGCTGTA
IL6 forward	AAGCCAGAGCTGTGCAGATGAGTA
IL6 reverse	TGTCCTGCAGCCACTGGTTC
PPARG forward	CACATTACGAAGACATTCCATTCAC
PPARG reverse	GGAGATGCAGGCTCCACTTTG
FOXO3 forward	GGTGCTAAGCAGGCCTCATCTC
FOXO3 reverse	AATGGCGTGGGATTCACAAAG
SOD1 forward	AGTGCAGGGCATCATCAATTTC
SOD1 reverse	CCATGCAGGCCTTCAGTCAG
MAP1LC3B forward	AGTTGGCACAAACGCAGGGTA
MAP1LC3B reverse	TTAGGAGTCAGGGACCTTCAGCA

### Statistical analysis

Data analysis of this study was conducted using R software (version 4.0.0). Charts were generated with GraphPad Prism 8.0 (GraphPad Software, CA, USA). Data are means ± standard error of measurement (SEM) and were compared via two-tailed Student’s *t*-tests, and *P* < 0.05 was regarded as statistically significant.

### Bioinformatics analysis workflow


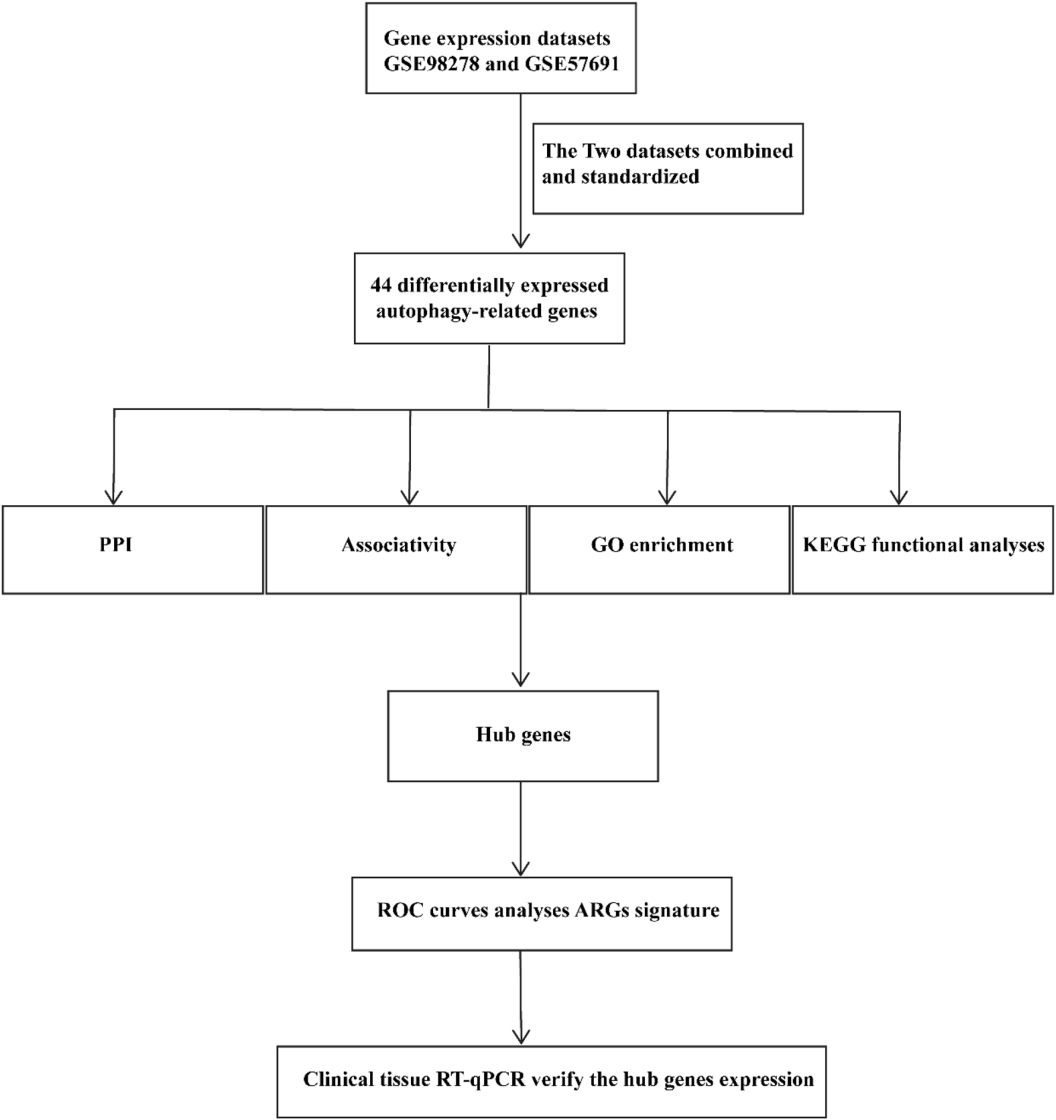


### DEARGs in AAA-Retrospective Analysis of ARGs

**Fig. 1:**
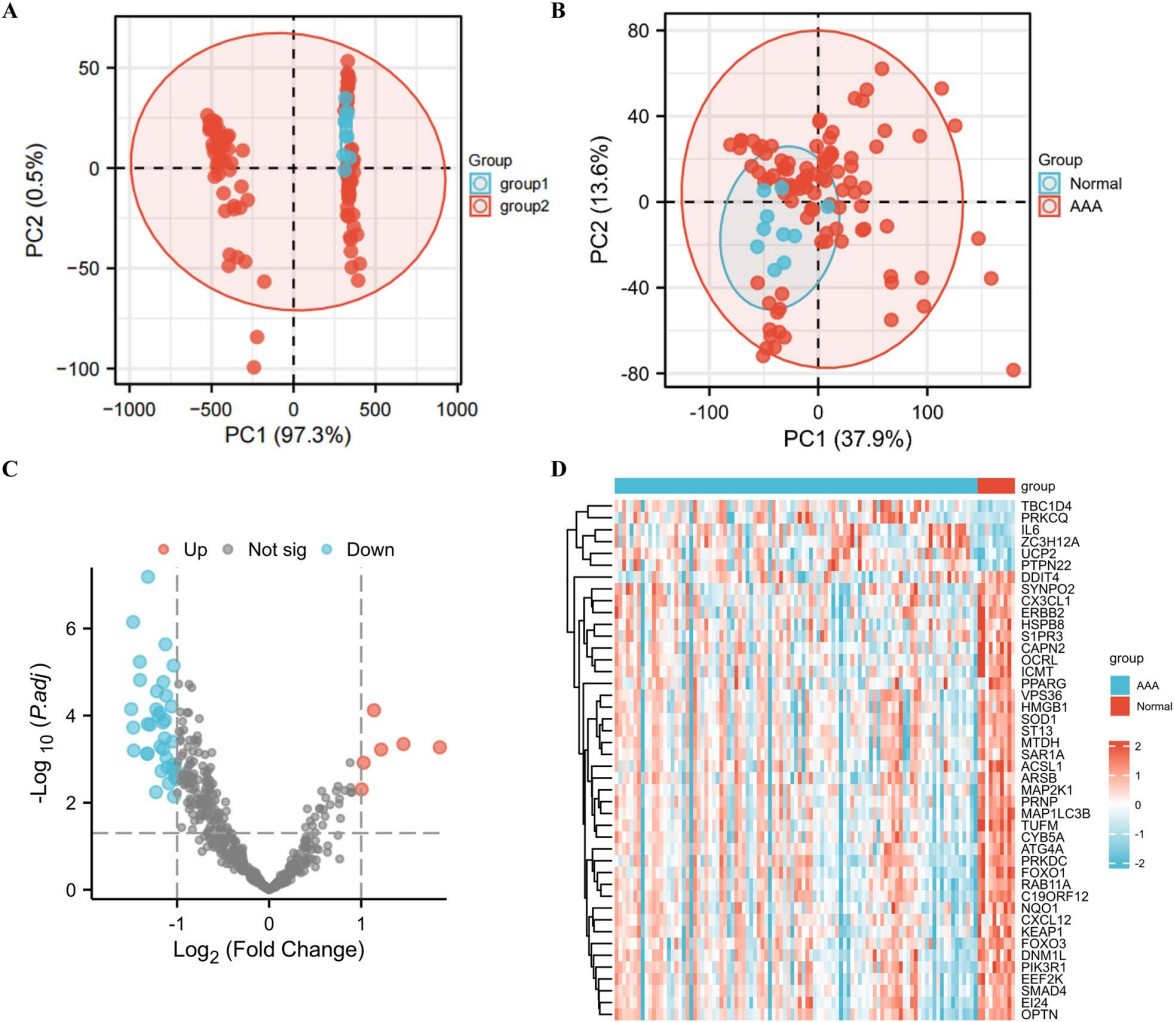
Differentially expressed autophagy-related genes in AAA and healthy samples. **A** Results of PCA before SVA. **B** Results of PCA after SVA. **C** Volcano plot of differentially expressed autophagy-related genes. Significantly up-regulated and down-regulated genes are represented by red and blue dots, respectively. Criteria used for the identification of differences: |log FC|> 0.5 and* P* < 0.05. **D** Heatmap of 44 differentially expressed autophagy-related genes in AAA and healthy samples. *AAA* abdominal aortic aneurysm, *FC* fold change, *PCA* principal component analysis

**Fig. 2:**
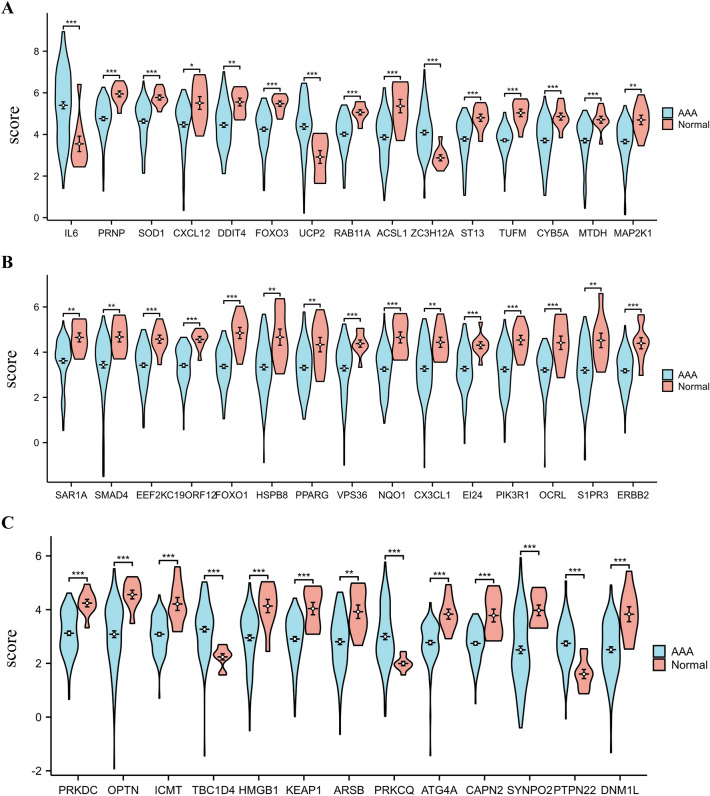
Violin diagram of 44 DEARGs in AAA and healthy samples. The “scores” on the Y axis represent relative gene expression. AAA patients and healthy examples, represented by green and red, respectively. *P < 0.05; **P < 0.01; ***P < 0.001

Initially, we downloaded GSE98278 and GSE57691 datasets from the GEO database. A total of 97 AAA patients and 10 healthy controls were included. SVA R was performed to eliminate batch effects between two different batch samples and verify the effect of batch correction by principal component analysis (PCA) (Fig. 1A, B). Subsequently, the LIMMA software package was performed to screen 44 genes (6 up-regulated genes and 38 down-regulated genes) from 796 ARGs using |log FC|> 1.0 and *P* < 0.05 as criteria (Table 4). The 44 DEARGs between the AAA and normal groups identified in the GSE98278 and GSE57691 databases are shown in volcano (Fig. 1C) and heatmap (Fig. 1D) plots. Figure 2A–C violin diagram shows the expression patterns of 44 candidate genes in normal and AAA samples. Among the six up-regulated genes, the expression changes of interleukin 6(IL-6), zinc finger CCCH-type containing 12A (ZC3H12A), protein kinase C theta (PRKCQ), protein tyrosine phosphatase non-receptor type 22 (PTPN22), uncoupling protein 2(UCP2), and TBC1 domain family member 4 (TBC1D4) were statistically significant. Of the down-regulated genes, prion protein (PRNP), superoxide dismutase 1 (SOD1), Acyl-CoA synthetase long-chain family member 1 (ACSL1), ST13 Hsp70 interacting protein (ST13), etc., displayed significant changes in expression.

**Table 4:** The 44 differentially expressed autophagy-related genes identified in AAA samples compared with healthy samples

Gene symbol	Gene full name	Log FC	*P*-value		Sign
IL6	Interleukin 6	1.854287696	0.000536548		Up
ACSL1	Acyl-CoA synthetase long-chain family member 1	− 1.498992684	7.11E−05		Down
FOXO1	Forkhead box O1	− 1.478291998	7.11E−07		Down
OPTN	Optineurin	− 1.475476971	0.000189003		Down
SYNPO2	Synaptopodin 2	− 1.470449509	0.000632708		Down
UCP2	Uncoupling protein 2	1.459662163	0.000450946		Up
MAP1LC3B	Microtubule-associated protein 1 light chain 3 beta	− 1.406036738	5.81E−06		Down
NQO1	NAD(P)H dehydrogenase, quinone 1	− 1.403057967	1.53E−05		Down
HSPB8	Heat shock protein family B (small) member 8	− 1.32606301	0.000744209		Down
S1PR3	Sphingosine-1-phosphate receptor 3	− 1.322071348	0.000759019		Down
DNM1L	Dynamin 1 like	− 1.319501904	0.000156247		Down
TUFM	Tu translation elongation factor, mitochondrial	− 1.316836492	6.54E−08		Down
PIK3R1	Phosphoinositide-3-kinase regulatory subunit 1	− 1.294565375	0.000163092		Down
SMAD4	SMAD family member 4	− 1.229514634	0.005736397		Down
FOXO3	Forkhead box O3	− 1.226609084	7.17E−05		Down
ERBB2	Erb-b2 receptor tyrosine kinase 2	− 1.219461333	2.78E−05		Down
ZC3H12A	Zinc finger CCCH-type containing 12A	1.217032418	0.000602389		Up
OCRL	OCRL inositol polyphosphate-5-phosphatase	− 1.204585369	0.00010173		Down
PRNP	Prion protein	− 1.189461334	8.64E−05		Down
HMGB1	High mobility group box 1	− 1.182508628	0.000513381		Down
CX3CL1	C-X3-C motif chemokine ligand 1	− 1.170147022	0.001883242		Down
EEF2K	Eukaryotic elongation factor 2 kinase	− 1.153423438	0.000144531		Down
CYB5A	Cytochrome b5 type A	− 1.15130727	0.000587073		Down
C19ORF12	Chromosome 19 open reading frame 12	− 1.150038744	1.70E−05		Down
PTPN22	Protein tyrosine phosphatase non-receptor type 22	1.139013995	7.56E−05		Up
SOD1	Superoxide dismutase 1	− 1.13450957	0.000333745		Down
KEAP1	Kelch-like ECH-associated protein 1	− 1.132006804	0.000127777		Down
ICMT	Isoprenylcysteine carboxyl methyltransferase	− 1.126722892	2.31E−06		Down
PRKDC	Protein kinase, DNA-activated, catalytic subunit	− 1.120876405	3.47E−05		Down
ARSB	Arylsulfatase B	− 1.116386777	0.000937924		Down
DDIT4	DNA damage-inducible transcript 4	− 1.106453053	0.001543176		Down
VPS36	Vacuolar protein sorting 36 homolog	− 1.090350128	0.003625821		Down
ATG4A	Autophagy-related 4A cysteine peptidase	− 1.05943196	6.17E−05		Down
RAB11A	RAB11A, member RAS oncogene family	− 1.048852339	0.00039715		Down
MAP2K1	Mitogen-activated protein kinase kinase 1	− 1.039636349	0.001667882		Down
CAPN2	Calpain 2	− 1.038225758	7.10E−06		Down
EI24	EI24 autophagy-associated transmembrane protein	− 1.03818983	0.001317094		Down
CXCL12	C-X-C motif chemokine ligand 12	− 1.034376299	0.007241633		Down
SAR1A	Secretion-associated Ras-related GTPase 1A	− 1.034002436	0.001859123		Down
TBC1D4	TBC1 domain family member 4	1.026985567	0.001213376		Up
PPARG	Peroxisome proliferator-activated receptor gamma	− 1.02321369	0.002583403		Down
ST13	ST13 Hsp70 interacting protein	− 1.020903612	0.001595684		Down
MTDH	Metadherin	− 1.008135363	0.002910239		Down
PRKCQ	Protein kinase C theta	1.004575424	0.004953416		Up

### Biofunctional enrichment analysis of the candidate ARGs

**Fig. 3:**
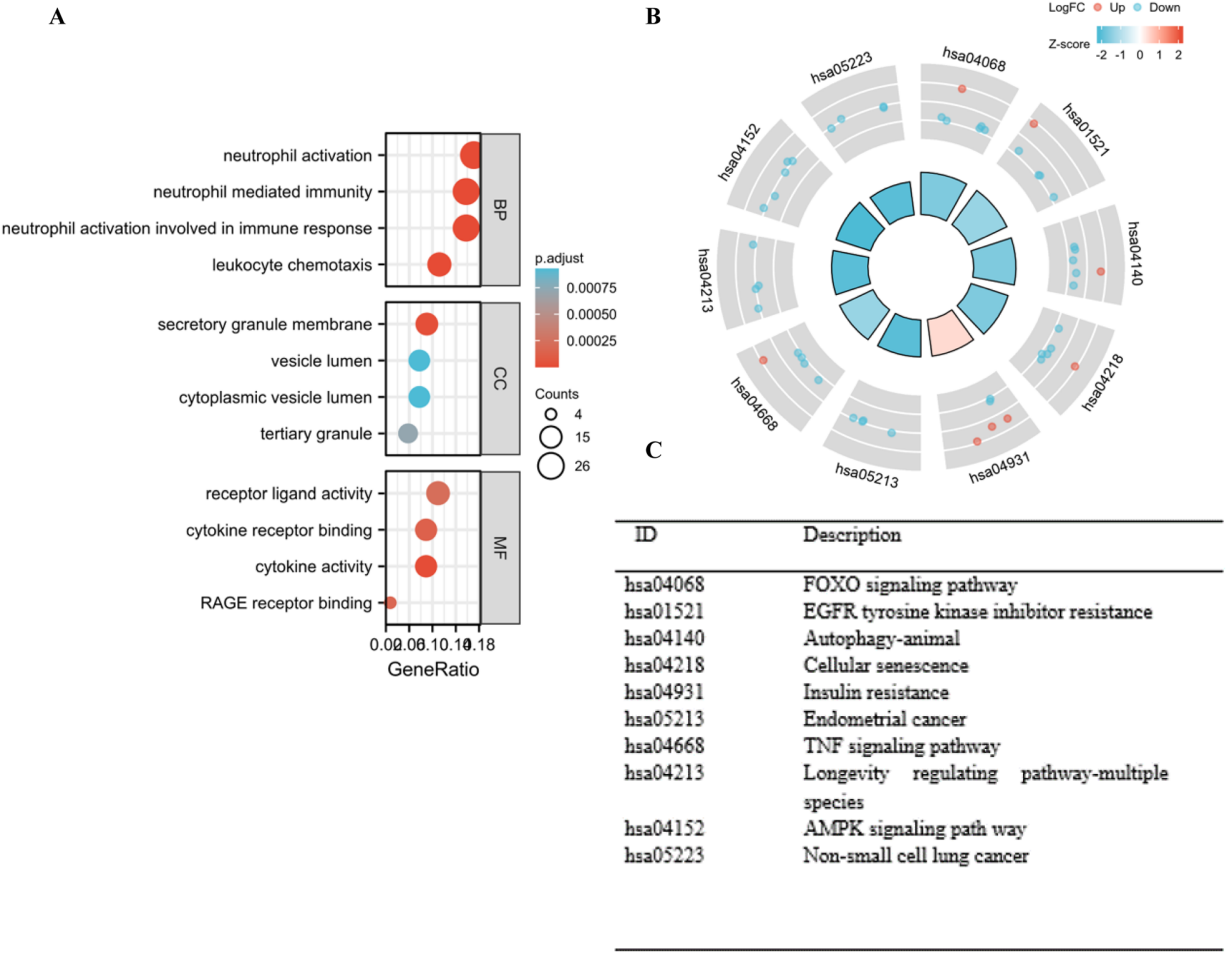
GO and KEGG enrichment analyses of 44 DEARGs. **A** Bubble diagram of GO enrichment term. **B**, **C**. KEGG enrichment analyses

The underlying biological functions of the 44 DEARGs were explored using GO and KEGG gene set functional analysis. The results of GO enrichment analysis presented that in terms of biologic processes these genes were considerably boosted in neutrophil activation, neutrophil intermediated immunity, neutrophil activation involved in immune response, and leukocyte chemotaxis. In terms of cellular components, the genes were enhanced in secretory granule membrane, vesicle lumen, cytoplasmic vesicle lumen, and tertiary granules. On the basis of molecular functions, the genes were markedly enriched in receptor ligand activity, cytokine receptor binding, cytokine activity, and RAGE receptor binding (Fig. 3A). KEGG enrichment analysis identified the candidate autophagy-related genes primarily involved in the FoxO signal pathway, EGFR tyrosine kinase inhibitor resistance, and autophagy (Fig. 3B, C).

### PPI network analysis and identification of hub genes of the candidate ARGs

**Fig. 4:**
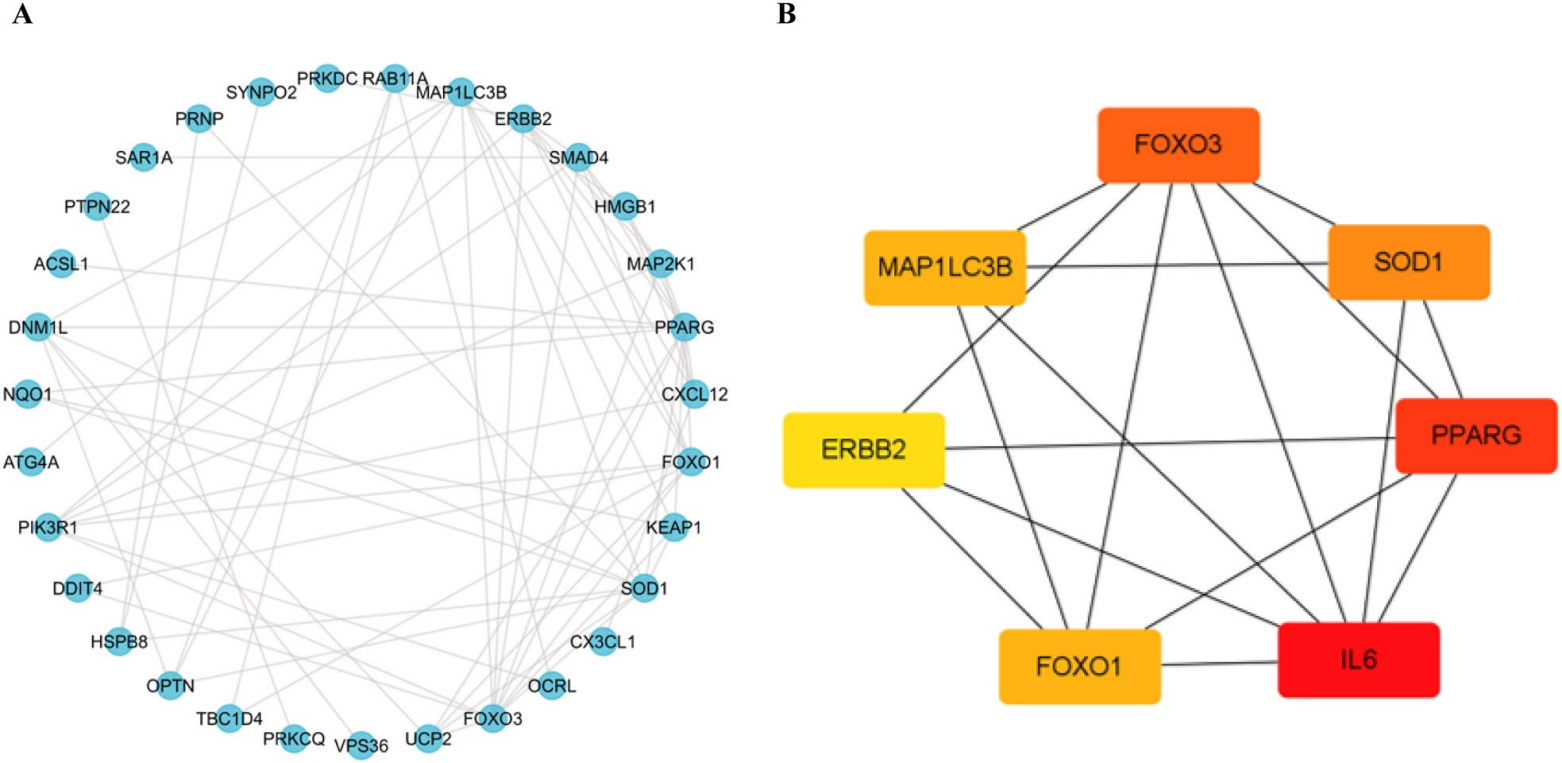
PPI analysis of candidate DEARGs. **A** PPI networks of candidate genes. **B** PPI subnetwork of the top 7 hub candidate genes. *PPI* protein–protein interaction

The PPI analysis demonstrated that candidate DEARGs interacted with each other and the candidate genes that did interact according to the predicted results were showed (Fig. 4A). In PPI network, the top seven scoring genes were identified as hub ARGs. Hub IRGs were IL6, PPARG, FOXO3, SOD1, MAP1LC3B, FOXO1, and ERBB2 (Fig. 4B).

### Performance of candidate ARGs signature

**Fig. 5:**
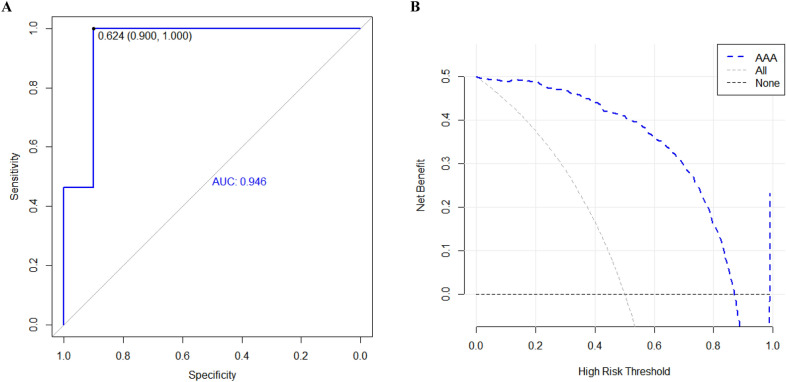
Testing of the model. DCA and ROC were performed to detect 7 hub genes. **A** ROC curves of ARGs signature. **B** DCA of the candidate autophagy-related gene signature. *DCA* decision curve analysis, *ROC* receiver operating characteristic

The receiver operating characteristic (ROC) curve was performed to explain that the logistic regression model established with 7 hub genes has excellent sensitivity for the diagnosis of AAA. The area under the curve of ARGs candidate genes was 0.946 (Fig. 5A); the higher the score, the higher the accuracy of the Logistic regression model built by 7 hub genes for predicting the process of AAA. DCA showed that within the best possible threshold probability area, ARGs signatures may be predictive of AAA (Fig. 5B).

This indicates that the Logistic regression model established using these 7 hub genes has clinical significance. However, the lack of comparability of existing clinical interventions makes it difficult to verify its potential advantages.

### Validation of the candidate ARGs in AAA clinical samples

**Fig. 6:**
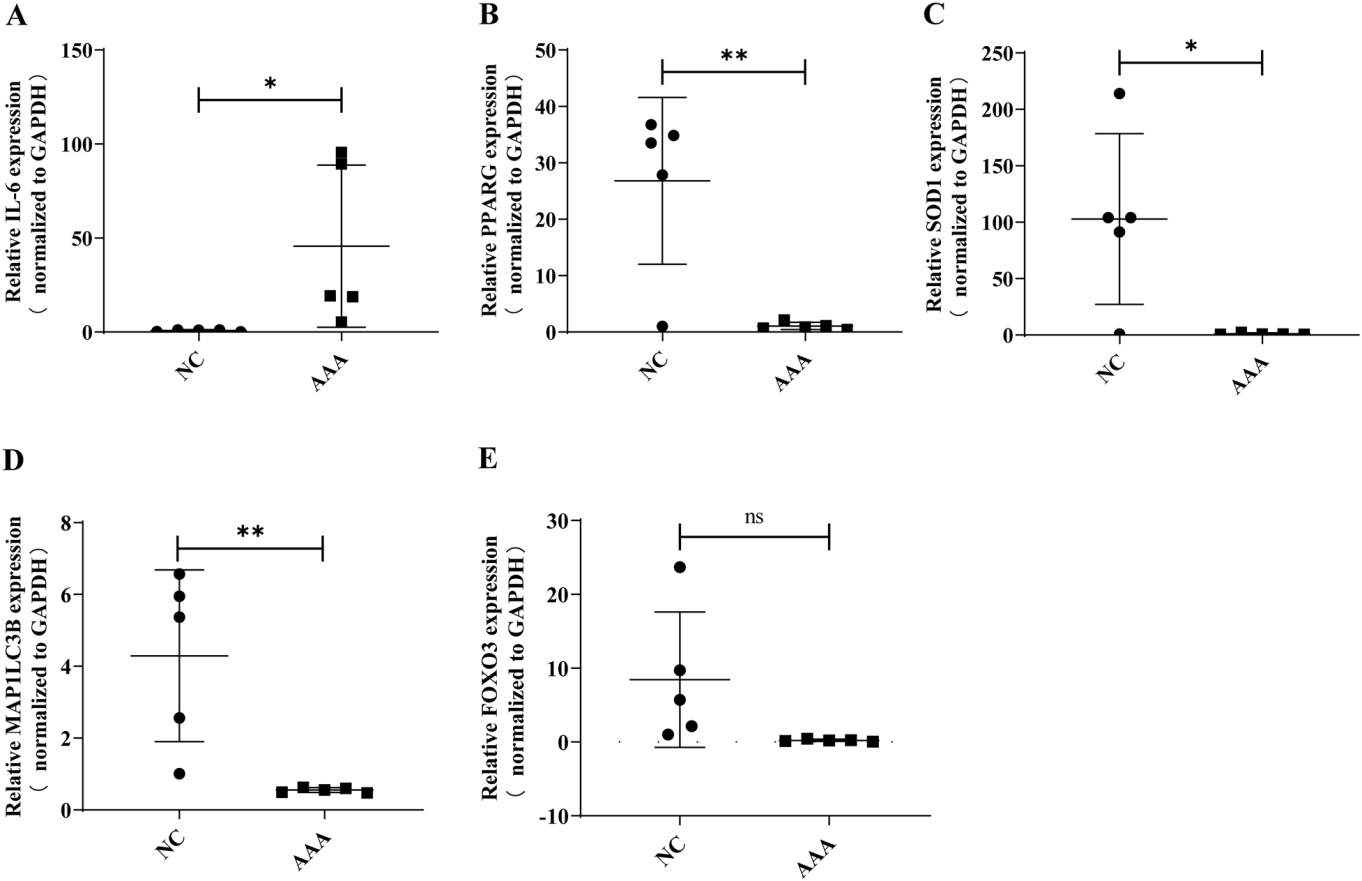
Verification of the expression of the top 5 hub genes in clinical samples. RT-qPCR was performed to detect the expression level of IL6, PPARG, FOXO3, SOD1, MAP1LC3Bn clinical samples. *P*-values were computed using Student’s *t*-test. *P < 0.05; **P < 0.01; ns, non-significant

To verify the results of bioinformatics analysis, we further identified the expression levels of the top five DEARGs in our clinical specimens using RT-qPCR (Table 2). Similar to the mRNA microarray results, IL6 was markedly elevated in AAA (Fig. 6A), while levels of PPARG.SOD1 and MAP1LC3B were appreciably decreased (Fig. 6B–D). However, no significant variation in the expression level of FOXO3 was discovered between the two groups (Fig. 6E).

## Discussion

AAA is a chronic vascular inflammatory disease and a vital reason of death from aortic rupture in adults, yet no effective clinical treatment is available. Risk factors for AAA include smoking, aging, inflammation, thrombosis, and atherosclerosis. Mounting evidence indicates that autophagy may have a critical role in AAA. For example, AAA risk factors are closely associated with autophagy [16–19], while Zheng et al. found that ATG is involved in the induce formation of autolysosomes in AAA tissues [20]. In addition, Li et al. showed that rapamycin, a powerful immunosuppressant, can inhibit the pathological process of AAA by inhibiting the mTOR pathway [21]. However, more evidence and further validation is needed to clarify the potential role of autophagy in the pathological mechanism of AAA.

Recently, with the development of next-generation sequencing, bioinformatics analysis has been extensively applied to explore and recognized latent biomarkers of some diseases [22], and many public databases have emerged, such as TGGA and GEO. The latest of a series of studies have explored the pathogenesis of AAA with potential therapeutic value in gene expression. Chen et al. constructed a co-expression network using WGCNA and analyzed gene components in abdominal aortic aneurysm and healthy control states. In their study, hub gene clusters (the most important clusters of the DEG co-expression network chosen by MCODE) including YIPF6, RABGAP1, ANKRD46, GPD1L, and PGRMC2 were identified [23]. These genetic factors have underlying diagnostic implications and may turn into biomarkers for abdominal aortic aneurysm. Moreover, Giusti et al. using expression profiles of microarray data showed that the autophagy gene ATG5 in peripheral venous blood of AAA patients was up-regulated compared with control patients [24]. Despite these advances, the understanding of pathogenesis and genetics of AAA is still not fully clarified. Consequently, it is essential to further discover new targets for the diagnosis and treatment of AAA.

As far as we know, several published articles have explored key genes and the role of autophagy in AAA. For example, a recent study reported on 10 hub genes in AAA, some of which are involved in chronic inflammation in patients [23]. However, bioinformatics analysis of ARGs in AAA remains indistinct. In this survey, we used bioinformatics analysis for the first time to identify 44 potential AAA-related ARGs from two GEO datasets (GSE98278 and GSE57691). One of the previous studies on ARGs of AAA confirmed that mutations in the IL-6-174G/C allele increased the risk of AAA development [25]. In addition, there is evidence that FOXO3a promotes phenotypic transformation of VSMS through the P62/LC3BII autophagy signal channel, accelerating the formation of AAA, and that reducing FOXO3a expression may stop AAA formation [26]. In the future, we plan to explore more subclinical ARGs associated with AAA.

This study used GO and KEGG enrichment analyses to elucidate the biological functions of DEARGs, which were mainly enriched in inflammatory cell activation, cell chemotaxis, the FoxO signaling path, autophagy, cellular senescence, TNF signal channel, longevity controlling pathway, AMPK signaling path, and non-small cell lung cancer. Previous evidence confirms that pathological traits of abdominal aortic aneurysm contain inflammatory disease, AS, and thrombosis [27]. In addition, macrophages, VSMS, and endothelial cells play a role. Autophagy plays a crucial part in all of these processes. For example, in ATG5-deficient ApoE−/− mice, autophagy absence causes hyperactivation of macrophage inflammation complex, accelerating plaque movement [28]. The latest study showed that P2RY12 receptor inhibits autophagy and reduces cholesterol effluence, promoting VSMC-derived foam cytogenic in advanced atherosclerosis. This suggests that P2RY12 receptor plays an important role in the regulation of macro-autophagy/autophagy and the formation of VSMC-derived foam cells in advanced atherosclerosis [29]. In addition, autophagy has been stated to play an essential role in bleeding and thrombotic diseases by regulating the count and function of platelet (PLT), which are the core factors of physiological hemostasia [30]. Therefore, it is necessary to conduct clinical or basic experiments to probe the underlying biological functions of these DEARGs.

This study identified 7 ARGs and found that five of these were distinctively expressed between the two groups. The lack of difference in FOX03 expression levels between groups may be due to large individual differences and small sample sizes and will be further explored and verified in the future. Previous evidence has identified several genes involved in cardiovascular disease. Nishihara et al. demonstrated that suppression of IL-6 can suppress Stat3 activation and expansion of AAA in mouse models [31]. Other studies have found that PPARG polymorphism is weakly associated with the development of AAA [32], and that PPARγ attenuates AAA by inhibiting inflammation and proteolytic degradation [33]. SOD1 has not been reported in AAA, but when treated with hydroxyl ethanol, it inhibits Ang II-induced Alzheimer’s disease, decreases the expressing of NF-κB, P65, TNF-α, and IL-1β, and increases the conveying of SOD1, MMP9, and GCLC in mice [34]. However, the precise mechanisms of these genes in AAA remain largely unknown and require further exploration. 

However, this study has some limitations. First, we obtained bioinformatics results from public chip data and did not obtain adequate clinical information. Second, the clinical sample size was small, and the results must be verified in a larger cohort. Third, this study validated the DEARGs in clinical specimens only and did not research the latent mechanisms of these genetic factors in AAA cells and animal models. Consequently, advanced exploration and investigation are needed in the future.

In conclusion, hub genes IL6, PPARG, SOD1, and MAP1LC3B may influence the onset and development of AAA by controlling autophagy. This study proposes a new characteristic of ARGs, enhances understanding of AAA, and may advance its diagnosis and therapy.

## Data Availability

Research submitted in the initial contributions includes Supplementary Material in the writing. Additional enquiries can be made to the corresponding authors.

## Abbreviations

AAA: Abdominal aortic aneurysm
GEO: Gene Expression Omnibus
PPI: Protein–protein interaction
GO: Gene ontology
KEGG: Kyoto Encyclopedia of Genes and Genomes
qRT-PCR: Quantitative real-time polymerase chain reaction
DEARGs: Differentially expressed autophagy-related genes
GEO database: https://www.ncbi.nlm.nih.gov/
SVA: Surrogate variable analysis
LIMMA: Linear models for microarray data
STRING database: https://www.string-db.org/
AS: Atherosclerosis
PLT: Platelets
BP: Biological process
CC: Cellular component
MF: Molecular function
